# Activatable Fluorophores for Imaging Immune Cell Function

**DOI:** 10.1021/acs.accounts.2c00070

**Published:** 2022-04-05

**Authors:** Lorena Mendive-Tapia, Marc Vendrell

**Affiliations:** Centre for Inflammation Research, The University of Edinburgh, EH16 4TJ Edinburgh, U.K.

## Abstract

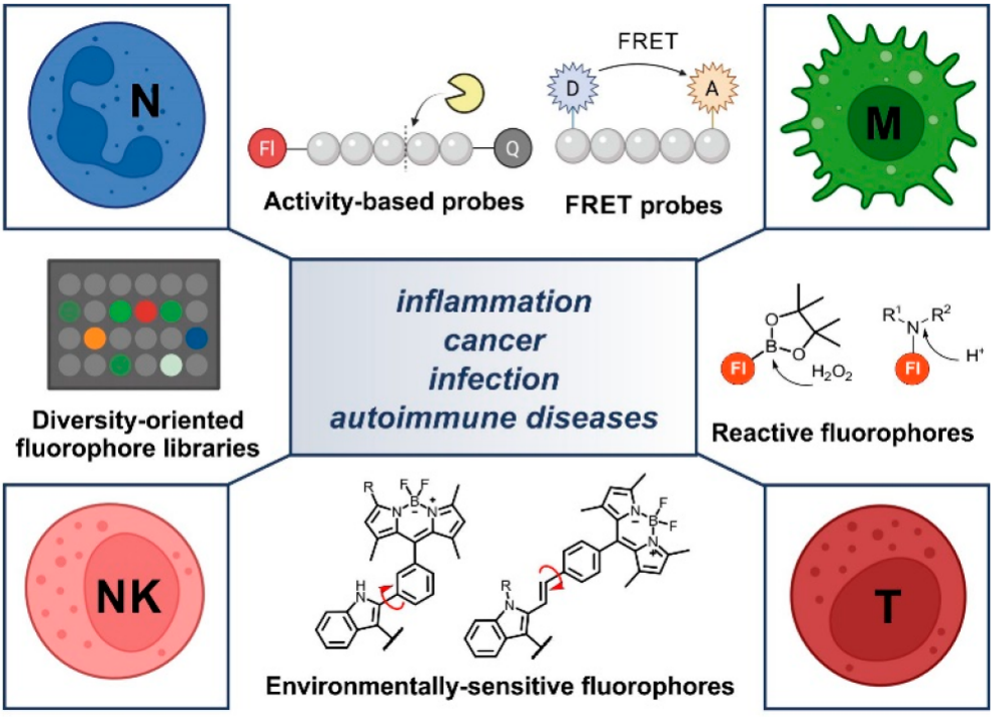

Optical imaging has become an essential tool to study biomolecular processes in live systems with unprecedented spatial resolution. New fluorescent technologies and advances in optical microscopy have revolutionized the ways in which we can study immune cells in real time. For example, activatable fluorophores that emit signals after target recognition have enabled direct imaging of immune cell function with enhanced readouts and minimal background. In this Account, we summarize recent advances in the chemical synthesis and implementation of activatable fluorescent probes to monitor the activity and the role of immune cells in different pathological processes, from infection to inflammatory diseases or cancer. In addition to the contributions that our group has made to this field, we review the most relevant literature disclosed over the past decade, providing examples of different activatable architectures and their application in diagnostics and drug discovery. This Account covers the imaging of the three major cell types in the immune system, that is, neutrophils, macrophages, and lymphocytes. Attracted by the tunability and target specificity of peptides, many groups have designed strategies based on fluorogenic peptides whose fluorescence emission is regulated by the reaction with enzymes (e.g., MMPs, cathepsins, granzymes), or through Förster resonance energy transfer (FRET) mechanisms. Selective imaging of immune cells has been also achieved by targeting different intracellular metabolic routes, such as lipid biogenesis. Other approaches involve the implementation of diversity-oriented fluorescence libraries or the use of environmentally sensitive fluorescent scaffolds (e.g., molecular rotors). Our group has made important progress by constructing probes to image metastasis-associated macrophages in tumors, apoptotic neutrophils, or cytotoxic natural killer (NK) cells against cancer cells, among other examples. The chemical probes covered in this Account have been successfully validated in vitro in cell culture systems, and in vivo in relevant models of inflammation and cancer. Overall, the range of chemical structures and activation mechanisms reported to sense immune cell function is remarkable. However, the emergence of new strategies based on new molecular targets or activatable mechanisms that are yet to be discovered will open the door to track unexplored roles of immune cells in different biological systems. We anticipate that upcoming generations of activatable probes will find applications in the clinic to help assessing immunotherapies and advance precision medicine. We hope that this Account will evoke new ideas and innovative work in the design of fluorescent probes for imaging cell function.

## Key References

- Fernandez, A.; Thompson, E. J.; Pollard, J. W.; Kitamura, T.; Vendrell, M.A Fluorescent Activatable AND-Gate Chemokine CCL2 Enables In Vivo Detection of Metastasis-Associated Macrophages. Angew. Chem. Int. Ed. Engl.2019, 58, 16894–168983153578810.1002/anie.201910955PMC6900180.^1^ Molecular AND-gate chemokines as one of the first examples of activatable probes to detect subpopulations of metastasis-associated macrophages in mouse models of cancer.
- Barth, N. D.; Subiros-Funosas, R.; Mendive-Tapia, L.; Duffin, R.; Shields, M. A.; Cartwright, J. A.; Henriques, S. T.; Sot, J.; Goni, F. M.; Lavilla, R.; Marwick, J. A.; Vermeren, S.; Rossi, A. G.; Egeblad, M.; Dransfield, I.; Vendrell, M.A Fluorogenic Cyclic Peptide for Imaging and Quantification of Drug-Induced Apoptosis. Nat. Commun.2020, 11, 40273278867610.1038/s41467-020-17772-7PMC7423924.^2^ Apo-15 as a highly stable fluorogenic peptide containing the environmentally sensitive reporter Trp-BODIPY to selectively stain apoptotic neutrophils in a mouse model of lung inflammation in vivo.
- Kaplanaris, N.; Son, J.; Mendive-Tapia, L.; Kopp, A.; Barth, N. D.; Maksso, I.; Vendrell, M.; Ackermann, L.Chemodivergent Manganese-Catalyzed C–H Activation: Modular Synthesis of Fluorogenic Probes. Nat. Commun.2021, 12, 33893409967210.1038/s41467-021-23462-9PMC8185085.^3^ Rational design of BODIPY-based fluorogenic probes to monitor changes in the membrane fluidity of T cells and their application in fluorescence-based screens to identify small molecule modulators of T cell activity.

## Introduction

In past decades, the scientific community has witnessed the remarkable progress of optical bioimaging technologies to facilitate in situ monitoring of molecular events in live cells with high spatiotemporal resolution. Recent improvements in the resolution power (e.g., super-resolution microscopy) and probe development (e.g., environmentally sensitive fluorophores, genetically engineered artificial proteins) have accelerated the chemical design of molecular reagents for imaging cellular activity in real time. Activatable fluorophores—which emit signals only after recognition and engagement with specific biomarkers (e.g., metabolites, enzymes, transporters)—display high signal-to-background ratios and enable direct imaging of biological systems without fixatives or washing steps. These constructs are coined as “activatable” or “smart” fluorophores because they are designed to elicit a distinguishable fluorescence readout under well-defined biochemical conditions. The preparation of activatable fluorophores involves expertise in multiple disciplines: from organic chemistry to synthesize “off-to-on” fluorescent structures to molecular and cell biology for the optimization and validation in relevant cells and model organisms.

**Figure 1:**
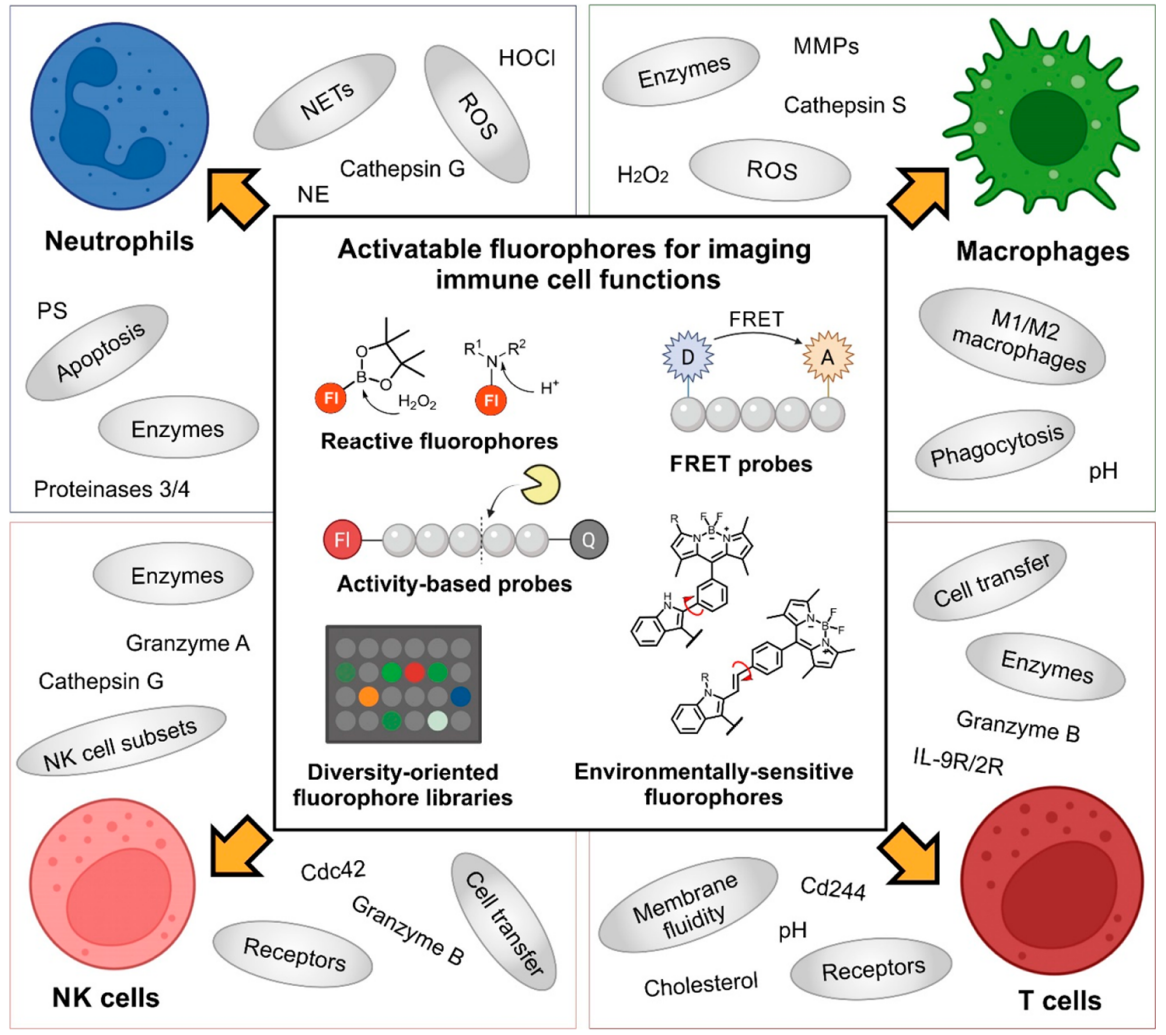
Chemical strategies for the visualization of immune cell function based on activatable fluorescent architectures. Smart fluorescent probes can be activated by different enzymes, cellular components, or environmental factors associated with the functional state of neutrophils, macrophages, NK cells, or T cells. Representative examples include the use of reactive fluorophores (e.g., H_2_O_2_, pH), probes employing Förster resonance energy transfer (FRET) mechanisms, activity-based fluorophores as enzyme substrates, diversity-oriented fluorescence libraries, or environmentally sensitive dyes. Abbreviations: NETs, neutrophil extracellular traps; ROS, reactive oxygen species; NE, neutrophil elastase; PS, phosphatidylserine; MMP, matrix metalloproteinases; D, donor; A, acceptor; F, fluorophore; Q, quencher. Images created with BioRender.com.

Immune cells are directly implicated in the progression of many diseases—from infectious and inflammatory diseases to cancer and neurological disorders—and therefore they are crucial to differentiate the physiological patterns of healthy and disease states. Many activatable fluorescent probes tracking immune cell function have been reported and linked to potential applications in diagnostics with the analysis of ex vivo samples^4^ and drug discovery by accelerating high-throughput screenings (Figure 1).^5^ In this Account, we will review the advances over the past 10 years in the synthesis and biological characterization of functional fluorophores targeting different immune cells, namely, macrophages,^6^ neutrophils, T cells, and NK cells.^7^ Whereas this Account covers the synthetic efforts behind the preparation of such chemical probes, it does not include many examples of genetically encoded reporters or immune-targeted nanomaterials, reviewed elsewhere.^8^

## Probes for Imaging Macrophages

Macrophages are multifunctional cells with essential roles in host defense, inflammation, and tissue repair, among others. Different chemical strategies for targeting macrophages have been reported, from the design of smart probes that release fluorescence signals after reaction with enzymes or intracellular mediators to the use of combinatorial libraries in cell-based screens.

### Enzyme-Activatable Fluorophores for Macrophages

Macrophages are important players in the progression and resolution of inflammation, with matrix metalloproteinases (MMPs) being key enzymes in many inflammatory responses. Some of the first chemical designs for imaging macrophage function in inflamed tissues were targeted toward MMP12.^9^ Activatable probes were prepared in the form of FRET enzyme substrates, where the PLGLEEA peptide sequence was flanked by fluorophores to produce ratiometric readouts after engagement with active MMP12. Among the different FRET pairs, coumarin343 and TAMRA exhibited the largest fold fluorescence increase. Notably, unlike other FRET probes, the authors used a palmitic acid moiety to anchor the reporter to the plasma membrane, allowing the detection of active MMP12 at the surface of activated macrophages and in bronchoalveolar lavages from mice undergoing lung inflammation.

Nonpeptidic structures have been also described to generate enzyme-activatable probes for macrophages.^10^ The infiltration of macrophages into tumors is correlated with poor clinical outcome, particularly anti-inflammatory M2 macrophages with high cathepsin activity. To target this family of enzymes, the activity-based probe BMV083 was reported as a cathepsin S targeting fluorophore combining a 1,4-disubstituted-1,2,3-triazole scaffold and the Cy5–QSY21 FRET pair. These druglike structures offered advantages for in vivo studies (e.g., proteolytic stability and bioavailability), and the authors employed BMV083 to visualize M2 macrophages in mouse tumors.

In addition to proteases, such as MMPs or cathepsins, other families of enzymes have been associated with active macrophages. Microglia are brain-specific macrophages, and they contribute to the progression of neural disorders. Kim et al. reported a structure–activity study to identify microglia-targeting fluorophores.^11^ In this case, the authors found that the monosubstituted BODIPY fluorophore CDr20 containing a 3-hydroxy-4-methoxystyryl moiety released bright fluorescence signals in microglia. Notably, the authors performed a genome-scale CRISPR-Cas9 knockout screen to identify the UDP-glucuronosyltransferase Ugt1a7c as the biological target responsible for the turn-on process through the glucuronidation of CDr20. This work represents an excellent example of how fluorogenic substrates for nonprotease enzymes can be used to selectively label subpopulations of macrophages.

### Intracellularly Activated Probes and Prodrugs

**Figure 2:**
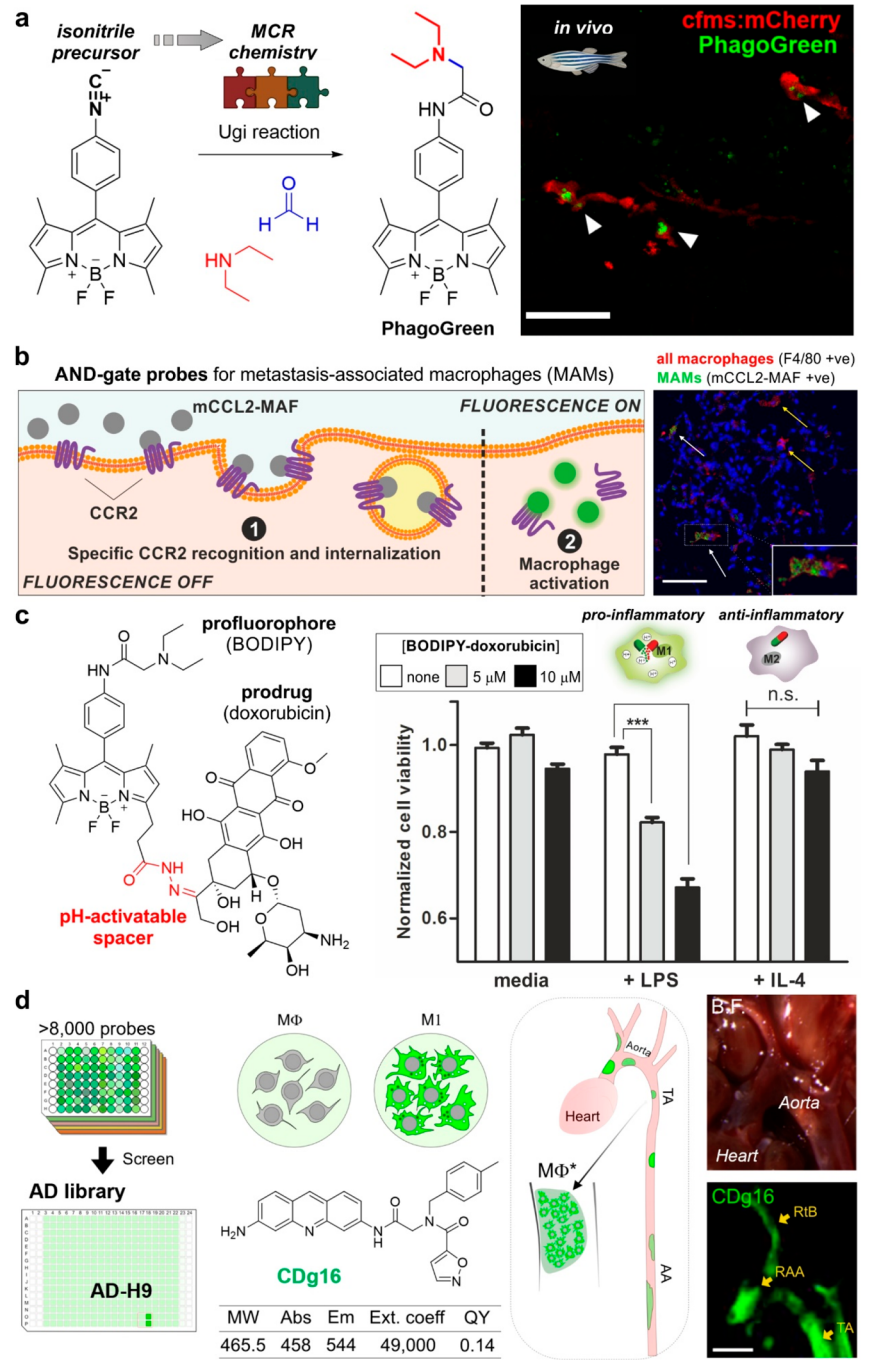
Chemical probes for imaging macrophage function. (a) Multicomponent reaction (MCR) chemistry using isonitrile groups to develop pH-activatable probes and representative in vivo imaging of transgenic zebrafish with mCherry-labeled macrophages after treatment with PhagoGreen (600 nM) for 30 min. The phagosomal localization of PhagoGreen is indicated with white arrowheads. Scale bar: 20 μm. (b) AND-gate probe (mCCL2-MAF) mechanism for CCR2+ metastasis-associated macrophages and representative confocal microscopy image of metastatic lungs. White and yellow arrows point at probe-stained MAMs and probe-unstained RMACs, respectively. Scale bar: 100 μm. (c) BODIPY–prodrug activatable conjugate and cytotoxicity after incubation in M1 and M2 mouse macrophages, showing a dose-dependent release of doxorubicin. (d) Diversity-oriented libraries for the selection of probe CDg16 and its application for the detection of atherosclerotic plaque areas by labeling of activated macrophages (MΦ*). Images show brightfield and fluorescence images of ApoE knockout (KO) mice. (a–c) Adapted from ref (12). Copyright 2013 American Chemical Society. Adapted from (14). Copyright 2017 American Chemical Society. From ref (1). CC BY 4.0. (d) From ref (19). CC BY 4.0.

In parallel to enzyme-activated probes, other approaches targeting intracellular mediators have been used for imaging macrophages. Our group designed a multicomponent reaction (MCR) strategy to generate PhagoGreen as the first BODIPY probe targeting the acidic phagosomes in active macrophages.^12^ PhagoGreen was selected from a collection of fluorophores that had been synthesized from a single isonitrile BODIPY precursor compatible with different MCRs (e.g., Ugi, Passerini, among others) (Figure 2a). The fluorescence emission of PhagoGreen was specifically blocked by bafilomycin A, an inhibitor of phagosomal acidification, and enabled imaging of phagocytic macrophages in vivo in zebrafish.

Subsequent efforts have been aimed at designing probes for defined subpopulations of macrophages. Our group pioneered the use of fluorescent chemokines (e.g., mCCL2-MAF) as activatable fluorophores for imaging metastasis-associated macrophages in tumors.^1^ The activatable chemokine mCCL2-MAF behaved as a molecular AND-gate with very low fluorescence background, emitting only after the chemokine ligand mCCL2 engaged with its cognate cell-surface receptor (i.e., the chemokine receptor CCR2 expressed in metastasis-associated macrophages) and subsequent intracellular activation in the acidic phagosomes. This original design allowed optical detection of metastasis-associated macrophages in mouse models of cancer for the first time (Figure 2b).

An additional feature of targeting intracellular biomolecules in macrophages is their potential application to release “caged” prodrugs that modulate cell function.^13^ Prodrug–fluorophore conjugates combining small molecules and pH-activatable fluorophores are unique tools to study pharmacological mechanisms in macrophages. Building on this concept, our group reported prodrugs to discern between M1 pro-inflammatory and M2 anti-inflammatory macrophages and to deplete only M1 macrophages without affecting neighboring cells (Figure 2c).^14^ In this case, the selective intracellular pH activation and drug release of a “caged” BODIPY-doxorubicin enabled the simultaneous fluorescence cell tracking and ablation of pro-inflammatory macrophages in a zebrafish model of acute inflammation, with the concomitant rescue of a proregenerative phenotype. Furthermore, ex vivo assays in human monocyte-derived macrophages demonstrated the translational potential of these chemical structures as immunomodulatory agents for inflammatory diseases. Given that macrophages can produce high levels of reactive oxygen species (ROS), recent chemical designs that can deploy cytotoxic drugs in disease environments with high levels of ROS and low pH^15^ are attractive tools for future approaches toward macrophage modulation.

### Intravital and In Vivo Imaging of Macrophage Function

A valuable application of immune-targeting fluorophores is their use for intravital imaging to examine complex biological mechanisms in vivo and with subcellular resolution. Several groups around the world have reported subcutaneous and mammary tumors via skin-fold chambers or optical windows to study tumor–macrophage interactions within primary and metastatic sites.^16^

Initial studies for imaging macrophages in tumors typically relied on genetically encoded fluorescent proteins (e.g., GFP, green fluorescent protein). For example, Harney et al. demonstrated that VEGFA signaling from TIE2-high macrophages caused local loss of vascular junctions, transient vascular permeability, and tumor cell intravasation.^17^ Importantly, the direct contact between macrophages, tumor cells, and endothelial cells has been correlated to metastasis in breast cancer patients.

The combination of optical windows with smart fluorescent probes has started to provide new insights into macrophage interactions that could help to design new therapeutic strategies. Maeda et al. designed the molecular probe pHocas for intravital imaging of osteoclasts, which are bone-resorbing cells that differentiate from macrophages.^18^ The pHocas probes combined bisphosphonates as osteoclast-targeting moieties with a pH-dependent fluorescence switch to detect actively resorbing cells with an acidic intracellular environment. Upon injection of pHocas probes into living mice, the authors performed time-lapse imaging and correlated osteoclast activity with changes in cell deformation and membrane fluctuations.

More recently, the group of Chang reported fluorophores for imaging activated macrophages in atherosclerotic plaques.^19^ The authors first performed an unbiased screening of a fluorophore library using LPS and IFNγ-treated macrophages as well as untreated cells to identify CDg16 as a fluorophore for active macrophages. Afterward, they demonstrated its application for labeling atherosclerotic plaques in mice using a fluorescent stereomicroscope (Figure 2d). CDg16 only showed strong signals in ApoE knockout mice, specifically in plaque areas, and subsequent studies identified the transporter Slc18b1 as the molecular target. Similar library screening approaches have been used to develop near-infrared (NIR) fluorophores, which exhibit advantages as in vivo optical reporters. Yoo et al. described a macrophage-targeting NIR probe for whole-body fluorescence imaging of macrophages in murine models of inflammation by LPS challenge and hind-limb ischemia with femoral artery ligation.^20^ The authors used intravital microscopy with Csf1r-EGFP transgenic mice and immunofluorescence staining with macrophage-specific markers CD68 and CD169 to corroborate the selectivity of the probe.

## Probes for Imaging Neutrophils

Neutrophils are the most abundant cells in peripheral blood, and they exhibit a broad range of immunomodulatory functions.^21^ Among the different fluorescent structures for monitoring their activity, we have classified them in probes for imaging active neutrophils, constructs targeting neutrophil extracellular traps, and fluorophores to label apoptotic neutrophils in inflamed tissues.

### Fluorescent Probes for Imaging Active Neutrophils

One of the main functions of neutrophils is the secretion of proteolytic enzymes that can destroy foreign bodies and/or pathogens. Like macrophages, fluorescent probes monitoring the activity of such enzymes are useful chemical tools for the detection and imaging of neutrophil function. Neutrophil elastase (NE) is one of the main enzymes secreted by neutrophils in infected and inflamed tissues, and it is directly implicated in the pathogenesis of acute and chronic inflammatory diseases. Gehrig et al. reported the fluorescent reporter NEmo-2 for as a ratiometric substrate for NE.^22^ The authors synthesized a FRET probe containing the peptide substrate sequence QPMAVVQSVPQ with a specific cleavage site between the valine residues tolerated by mouse and human NE. The lipidation of NEmo-2 with palmitic acid enabled monitoring enzymatic activity at the surface of neutrophils in a mouse model for lung inflammation, whereas the lipid-free analogue showed no activity in the lung fluid. Following designs aimed at increasing the selectivity and signal-to-noise ratios. Avlonitis et al. described a tribranched fluorescein-labeled construct of NE-reactive sequences (APEEIMRRQ) that were internally quenched due to the proximity of the fluorophores before reaction with the enzyme.^23^ With this construct, the authors achieved rapid and highly specific measurements of NE activity (e.g., over proteinase 3 and cathepsin G (CatG)) in primary human neutrophils.

**Figure 3:**
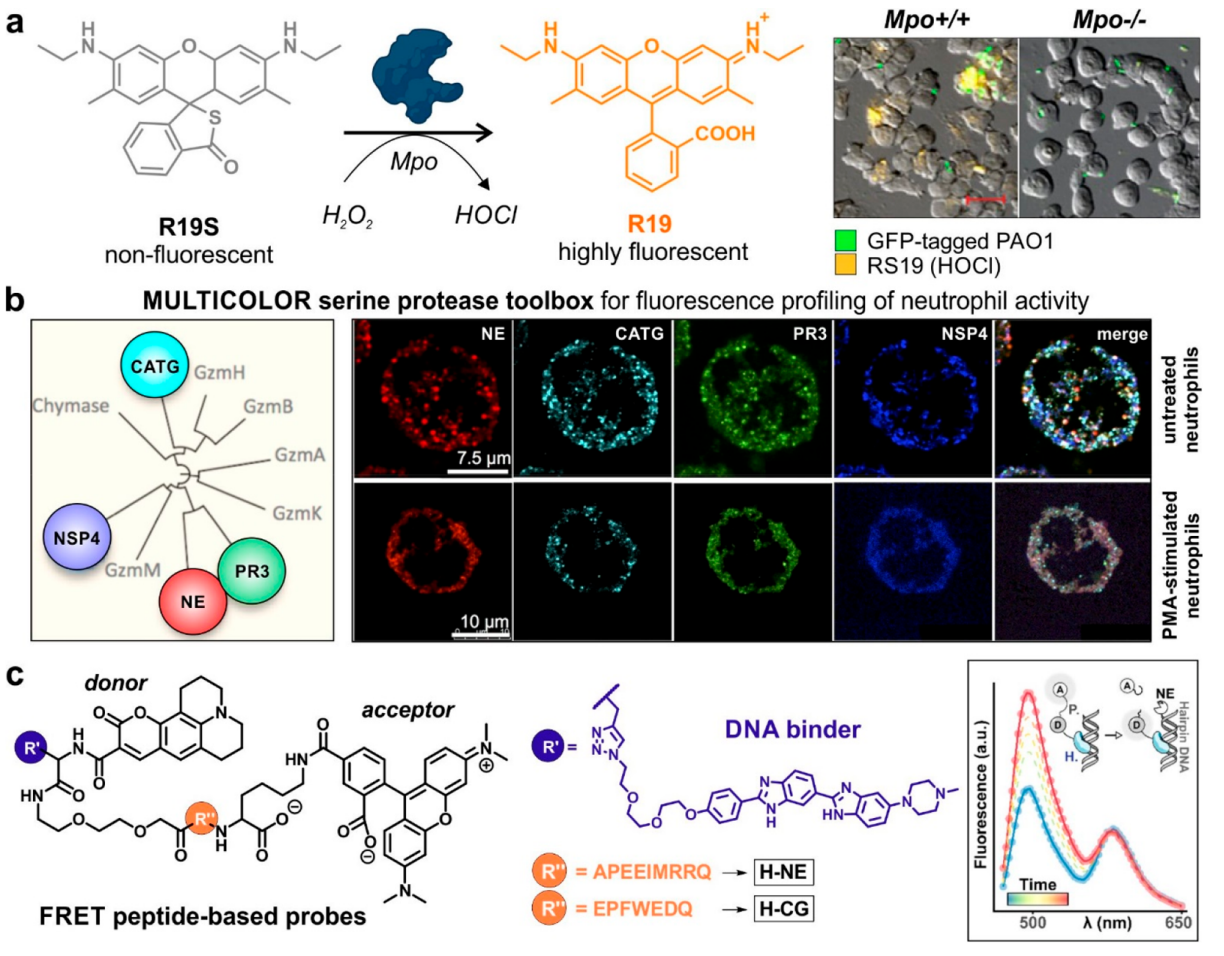
Activatable reporters to study neutrophil activity. (a) HOCl-activatable fluorophore R19S for monitoring active neutrophils and confocal microscopy images of neutrophils derived from Mpo+/+ or Mpo–/– mice after treatment with GFP-tagged PAO1 (green) and R19S (orange) for 1 h. Scale bar: 20 μm. (b) Multicolor toolbox of serine protease-targeting probes for multiplexed neutrophil imaging. Representative confocal microscopy images of the localization of active NSPs in neutrophil granules treated or untreated with PMA for 3 h. Images acquired under excitation with 488, 552, and 648 nm lasers. (c) Probes for neutrophil extracellular traps based on DNA minor groove binders and H-NE and H-CG probes. Representative example of the H-NE (2 μM) fluorescence signal increase, recorded for 15 min after the addition of NE (1 nM). (a) From ref (24). CC BY 4.0. (b) Adapted from ref (25). Copyright 2017 American Chemical Society. Adapted from ref (30). Copyright 2020 American Chemical Society.

In addition to NE, neutrophils use a wide range of biomolecules to action innate immune defense. For instance, neutrophils generate reactive oxygen species, such as hypochlorous acid (HOCl), to kill invading pathogens during infection. In neutrophils, HOCl is mostly generated by the enzyme myeloperoxidase (MPO), which converts hydrogen peroxide into HOCl. The group of Yoon developed highly sensitive fluorophores to detect HOCl in living cells and organisms.^24^ Among these, the rhodamine-based probe R19S was prepared from a commercially available rhodamine 6G in only two steps and demonstrated high selectivity for HOCl over other reactive oxygen species. Notably, the quenched thioester of R19S rapidly reacts with HOCl, which leads to the replacement of the sulfur atom by an oxygen atom and concomitant opening of the lactone ring to emit fluorescence (Figure 3a).

The myriad of enzymes that are present in neutrophils has also opened the possibility to design multicolor probes to simultaneously monitor the activity of different proteases. In this context, the work by Kasperkiewicz et al. is one remarkable example.^25^ The authors synthesized a toolbox of activity-based probes targeting four serine proteases (i.e., NE, CatG, proteinases 3 and 4). Enzyme recognition sequences contained natural and unnatural amino acids to enhance selectivity and were labeled with multiplexable fluorophores (e.g., BODIPY-FL, Cy3, Cy5, and Cy7) that emit at nonoverlapping wavelengths. Notably, the resulting toolbox was used in fluorescence microscopy experiments to image all four enzymes and to analyze their distribution in the azurophil granules of nonactivated and PMA-stimulated neutrophils, gaining new insights into how the activity of these proteins influences neutrophil activity (Figure 3b).

More recently, novel chemical approaches have been designed to generate fluorescent reporters of metabolic activity in neutrophils. Our group developed SCOTfluors as one of the smallest family of fluorophores for labeling small-molecule metabolites without impairing their normal uptake in live neutrophils.^26^ Lactic acid is an essential metabolite in neutrophils, particularly under low-oxygen hypoxic conditions. The conjugation of a red fluorescent SCOTfluor to l-isoserine was used to generate the first fluorescent analogue of lactic acid and to study its transport in live cells. Notably, this probe allowed flow cytometry analysis to observe increased uptake of lactic acid in hypoxic (i.e., 1% O_2_) versus normoxic (i.e., 20% O_2_) environments.

Selective imaging of neutrophils has been also recently achieved by targeting other metabolic routes, such as lipid biogenesis.^27^ Gao et al. reported highly lipophilic fluorescent probes for selective targeting of human neutrophils over other blood cells. The authors screened collections of fluorescent fatty acids in white blood cells to identify NeutropG as a neutrophil-targeting BODIPY-based probe. Furthermore, experiments were performed to demonstrate that NeutropG entered neutrophils through the cell-surface receptor CD36 and concluded that its selectivity was driven by the metabolic enzymes ACSL1 and DGAT2, which are directly implicated in lipid droplet formation.

### Probes for Imaging Neutrophil Extracellular Traps

One notable feature of neutrophils is their ability to form weblike chromatin structures called neutrophil extracellular traps (NETs). In the past decade, NETs have been directly related to different aspects of neutrophil biology. For instance, Albrengues et al. discovered that the formation of NETs during sustained inflammation awakened dormant tumor cells to aggressively grow lung metastases in mouse models of cancer.^28^ More recently, higher amounts of NETs have been found in hospitalized COVID-19 patients receiving mechanical ventilation as compared to hospitalized patients breathing room air.^29^

With the aim of targeting these extracellular structures, the group of Schultz reported chemical tools to study protease activity in NETs with high spatiotemporal resolution.^30^ NE, cathepsins, and MMPs are proteases commonly found in NETs. The authors exploited this feature to prepare FRET peptide-based enzymatic reporters armed with Hoechst 33258 as a DNA binder to monitor the activity of DNA-bound NE and CatG (Figure 3c). Interestingly, the study concluded that NE but not CatG maintained catalytic activity in NETs and high levels of protease activity were found in sputum samples from cystic fibrosis patients. Subsequently, Rios et al. reported a fluorescein-based dendrimer structure to track NE activity in NETs.^31^ In this case, the combination of proximity-based and Dabcyl-based quenching mechanisms led to constructs with very low background signals and enhanced fluorescence fold increases. Finally, fluorescent probes binding to extracellular DNA have been also successfully used for imaging NETs.^32^

### Fluorescent Probes for Imaging Neutrophil Apoptosis

Neutrophils play important roles in inflammation and tissue repair because the deficient clearance of apoptotic neutrophils by phagocytes contributes to the exacerbation of many autoimmune and inflammatory diseases. To this end, several assays to assess neutrophil apoptosis (e.g., DNA-binding dyes, annexin proteins, or caspase-targeting probes) and phagocytic clearance in vitro have been reported.^33^

**Figure 4:**
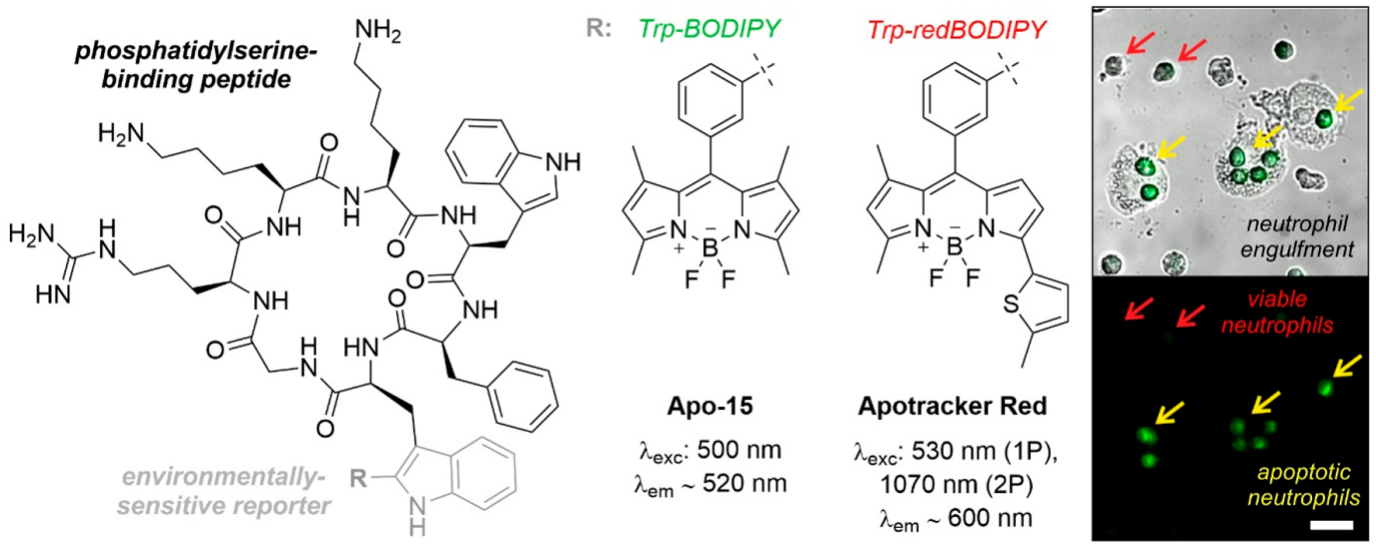
Apo-15 and ApoTracker Red as probes for in vivo imaging of apoptotic neutrophils. Representative brightfield and fluorescence microscope images of Apo-15-treated human neutrophils (green) in coculture with human monocyte-derived macrophages (MDMs). Red arrows identify Apo-15-negative viable neutrophils, and yellow arrows indicate MDMs that have engulfed Apo-15-labeled apoptotic neutrophils. Scale bar: 20 μm. From ref (2). CC BY 4.0.

Our group contributed to this field with the rational design of Apo-15 as a highly stable fluorogenic peptide to selectively stain apoptotic neutrophils in vitro and in vivo.^2^ Apo-15 binds to phosphatidylserine, which is exposed on the surface of apoptotic cells but not in viable cells, and uses the environmentally sensitive Trp-BODIPY amino acid^34,35^ as a fluorogenic reporter for wash-free imaging (Figure 4). Notably, unlike annexins, Apo-15 can label apoptotic neutrophils in a calcium-independent manner, and we demonstrated its application for the quantification and imaging of LPS and drug-induced neutrophil apoptosis in mouse models of lung inflammation. Furthermore, we addressed some of the optical limitations of Apo-15 (e.g., short emission wavelength) with the design of red-emitting analogues that incorporate Trp(redBODIPY)^36^ as a highly fluorogenic amino acid with emission of >600 nm. The resulting Apotracker Red shows enhanced signal-to-noise ratios and good compatibility for multiphoton intravital imaging of apoptotic cells in vivo (Figure 4).^37^

## Probes for Imaging T Cells and NK Cells

T cells and natural killer (NK) cells play pivotal effector functions in diverse defense mechanisms against infected or cancer cells, releasing cytokines and enzymes upon activation. Therefore, the tracking of recruitment, viability, and activation of T and NK cells is important to assess the efficacy of cell-based immunotherapies.

### Probes for Monitoring NK Cell Function

The response of NK cells after the formation of immunological synapses with target cells depends on cytoskeletal rearrangements and motility processes. Cdc42 is a Rho GTPase that coordinates vesicular transport and the formation of sensory contact domains (e.g., filopodia) during cell-to-cell interactions. The activity of Cdc42 in human NK cells was successfully imaged with a FRET biosensor (i.e., Raichu-Cdc42) expressing a monomeric red fluorescent protein (mRFP) and enhanced green fluorescent protein (eGFP) for fluorescence lifetime imaging microscopy (FLIM).^38^ Carlin et al. demonstrated that Cdc42 exhibited a periodic and oscillating activity during NK cell–target cell interactions, unlike in T cells, and identified the Akt and p85α subunits of phosphoinositide 3-kinase (PI3K) as key regulators of the immunological synapse.

Recent strategies to assess NK function have focused on enzyme-activatable probes. Penczek et al. described the synthesis of an activity-based probe MARS116 reporter for CatG, a serine protease found on the cell surface of NK cells.^39^ MARS116 contained a biotinylated peptide and a phosphonate warhead group to react with CatG and distinguish NK cell subsets characterized by different CD16 and CD56 expression levels. The authors concluded that CatG was present on CD16^–^CD56^dim^ cells, CD16^dim^CD56^–^ cells, and CD16^bright^CD56^–^ cells but it was only proteolytically active in the former two cell populations, suggesting that the activity of CatG is different in various NK cell subsets. NK cells promote apoptosis in target cells (e.g., cancer cells) through the secretion of perforins and serine proteases such as granzymes, being granzyme A (GrzA) and granzyme B (GrzB) the most common ones. Kolt et al. reported a selective probe targeting GrzA, whereby the authors first screened a peptide library to identify an optimal protease sequence and later coupled it to a fluorescent Cy5 moiety and a diphenyl phosphonate warhead.^40^ Unlike anti-GrzA antibodies, the probe only reacted with the active enzyme in human NK92 cells and the covalent warhead enabled detection of the labeled enzyme by SDS-PAGE analysis of cell lysates.

**Figure 5:**
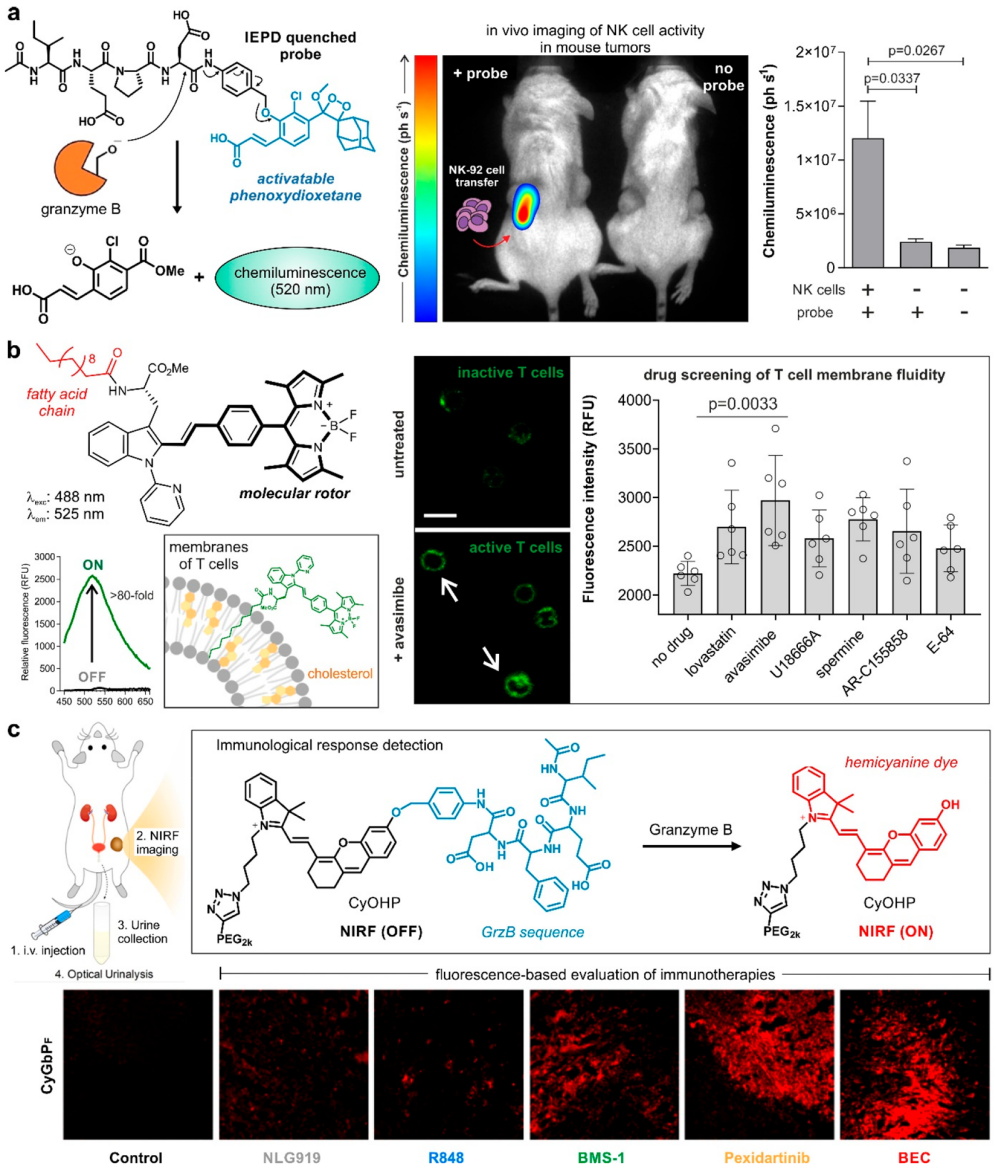
Optical reporters to study NK cells and T cells. (a) Chemiluminescent GrzB-activatable probe conjugated to a phenoxydioxetane reporter for the in vivo imaging of NK cell activity in tumor-bearing mice. Only the right tumor (red arrow) was injected with NK-92 cells with the left tumor being NK cell free. (b) BODIPY-based fluorogenic probe to detect cholesterol fluctuations in T cells and screening of small molecule modulators of CD8+ T cells. Fluorescence confocal microscopy images showing the staining (1 μM, green) of control CD8+ T cells and CD8+ T cells activated with avasimibe (30 μM). White arrows point at the plasma membrane localization of probe. λ_exc_/_em_, 488/525 nm; scale bar, 10 μm. (c) GrzB-activatable CyGbP_F_ for in vivo real-time NIRF imaging of immune responses in living mice. Representative confocal fluorescence images of tumor sections of immunotherapeutics-treated mice at the end of real-time tracking. Immunotherapeutics and CyGbPF (red) were intravenously administered. (a and b) From (42). CC BY 4.0. From ref (3). CC BY 4.0. (c) Adapted from ref (48). Copyright 2020 American Chemical Society.

The group of Kasperkiewicz used an analogous approach to synthesize activatable probes for GrzB.^41^ Using a combinatorial library approach to optimize substrate specificity, the authors synthesized a turn-on probe bearing Cy3 and BHQ2 as the fluorophore–quencher pair. The fluorogenic construct allowed real-time imaging of GrzB activity in live NK-like YT cells but not in other cell lines lacking active GrzB. The authors suggested active GrzB is constitutively released in NK-like YT cell lines in the absence of target cell engagement. More recently, our group designed the first chemiluminescent probe to image in vivo the killing capacity of NK cells against cancer cells.^42^ A GrzB-specific substrate was conjugated to an activatable phenoxydioxetane reporter via a self-immolative linker to release rapid and selective chemiluminescence upon cleavage of the substrate (Figure 5a), with 2 orders of magnitude higher signal-to-noise ratios than the commercial aminomethylcoumarin fluorescent reagents. Notably, the probe was used in tumor-bearing mice that underwent NK cell transfer, highlighting the potential of such probes to assess the efficacy of immunotherapies in preclinical models.

### Fluorescent Probes for Imaging T Cell Activity

A number of strategies to study T cell function have focused on the design of chemical tools for imaging the distribution and composition of cell membrane components. As an example, our group recently reported the rational design of BODIPY-based fluorogenic probes to monitor changes in the membrane fluidity of T cells as direct reporters of their activity.^3^ These activatable probes were based on molecular rotors that emitted strong fluorescence in response to the cholesterol found in the membranes of T cells (Figure 5b). The probes contained a fatty acid chain to facilitate anchoring to the plasma membrane and were used in a fluorescence-based screen to identify small molecule modulators of Jurkat T cells and validate the findings in primary human CD8+ T cells. In a related approach, Kwon et al. applied a combinatorial lipid-oriented library to distinguish T cells from B cells and identified a B cell selective probe (CDgB).^43^ Notably, the authors used CDgB to selectively label B cells in mixtures of splenocytes without the help of antibodies. In another example, fluorescently labeled antibodies were implemented in FRET imaging studies to investigate the cell surface organization of interleukin-9 and interleukin-2 receptors (IL-9R and IL-2R, respectively), which have a crucial regulatory role in T cells.^44^ Positive FRET measurements revealed that IL-9R accommodates at the surface of human T lymphoma cells in spatially segregated domains or in association with IL-2R and MHC glycoproteins, which may affect the assembly and signaling capability of these receptors.

Other approaches have been also employed to generate probes that can emit detectable signals inside T cells. Pacheco et al. monitored dynamic changes in the expression of the CD244 receptor on antiviral CD8+ T cells via the complexation of biotinylated anti-CD244 Ab to a pH-sensitive fluorophore–avidin conjugate.^45^ Our group reported the preparation of N-substituted tricarbocyanine dyes (CIR38) to visualize the long-term accumulation of T cells in lymph nodes in vivo.^46^ The *N*-triazole substitution conferred improved photostability and cell permeability to the tricarbocyanine core, overcoming the limitations of other NIR agents. In a mouse model of T cell activation, the administration of CIR38-labeled CD4+ T cells was followed by whole-body NIR fluorescence imaging, which allowed tracking of small numbers of cells up to 7 days with no leakage to other cell populations.

Granzymes are major effectors of cytotoxic T cells and have been targeted in macromolecular designs to measure GrzB activity in T cells. Konishi et al. prepared a polylysine graft copolymer including the GrzB-substrate IEPD peptide for in vivo imaging of T cell mediated injury in mouse models of acute and chronic myocarditis.^47^ The emission signals of the probe correlated with the severity of myocarditis and were used to monitor the efficacy of anti-inflammatory drugs (e.g., dexamethasone). In a related strategy, the group of Pu designed responsive GrzB probes that conjugated the IEPD sequence to NIR hemicyanine dyes through a polyethylene glycol (PEG) moiety to enable passive targeting to tumors from systemic circulation.^48^ Remarkably, these constructs enabled the longitudinal evaluation of immunotherapies in live mice (Figure 5c). Furthermore, because a major fraction of the probes was excreted through the kidneys within 24 h postinjection, the authors applied this technology for analysis of cleaved GrzB in urine samples. More recently, the group extended the range of GrzB-activatable probes with dual NIR and photoacoustic (PA) reporters for in vivo imaging of immune activation.^7^ In this case, the authors monitored both NIR fluorescence and PA signals to generate ratiometric readouts associated with the levels of active GrzB found in the tumors of living mice.

## Conclusions and Outlook

Multiple chemical strategies have been described for the synthesis and optimization of immune-targeted smart fluorophores. The diversity of chemical structures and activation mechanisms within this molecular toolbox is outstanding, from receptor-mediated probes and environmentally sensitive peptides to enzyme-triggered FRET sensors. Regarding the first two types, approaches to incorporate optical reporters with enhanced tissue penetration (e.g., fluorophores emitting in the NIR-II window, PA probes) as well as fluorogens into supramolecular sensing structures (e.g., noncanonical building blocks for genetic engineering and/or solid-phase peptide synthesis^49,50^) will open up a whole range of opportunities for immune sensing, including key subpopulations of immune cells in diseased environments. With regard to the latter, the design of combinatorial libraries will accelerate the identification of disease biomarkers^51^ and new fluorophores targeting enzymes other than proteases (e.g., glycosidases) will help researchers analyze the multiple role(s) of immune cells in different biological systems.

Regardless of targets and structures, one important aim for research groups developing immune-targeted fluorophores will be their translation to the clinic. Several examples have demonstrated the potential value of activatable fluorophores for imaging in humans, either to target macrophages in early stage cancer lesions^52,53^ or to monitor neutrophil function in the lungs of patients with acute respiratory distress syndrome.^54^ The adaptation and fine-tuning of these chemical innovations into healthcare technologies and clinical environments will create a new generation of sophisticated tools to accelerate precision medicine and interrogate biology under physiological conditions.
